# Deep hematologic response to RD treatment in patients with multiple myeloma is associated with overexpression of IL-17R in CD138+ plasma cells

**DOI:** 10.1038/s41598-024-74558-3

**Published:** 2024-10-09

**Authors:** Piotr Kulig, Karolina Łuczkowska, Bogusław Machaliński, Bartłomiej Baumert

**Affiliations:** 1 Department of General Pathology, Pomeranian Medical University, 70-111 Szczecin, Poland; 2 Pharmaceutical Facility of Pomeranian Medical University, 71-899 Szczecin, Poland; 3 Department of Hematology and Transplantology, Pomeranian Medical University, 71-252 Szczecin, Poland

**Keywords:** Multiple myeloma, Lenalidomide, IL-17, Good response to therapy, Tumor niche, Immune response, Bone marrow microenvironment, Myeloma, Cancer, Cancer microenvironment

## Abstract

Lenalidomide (LEN) is widely used immunomodulatory drug (IMiD). Nonetheless, despite its efficacy, over time patients become resistant to LEN and relapse. Due to high clinical relevance, drug resistance in MM is being thoroughly investigated. However, less is known about predictors of good response to LEN-based treatment. The aim of this study was to identify molecular pathways associated with good and long response to LEN. The study included newly diagnosed MM patients (NDMM) and MM patients treated with first-line LEN and dexamethasone (RD) who achieved and least very good partial remission (VGPR). RNA was isolated from MM cells and new-generation sequencing was performed. Obtained results were validated with qRT-PCR. A global increase in gene expression was found in the RD group compared to NDMM, suggesting the involvement of epigenetic mechanisms. Moreover, upregulation of genes controlling the interaction within MM niche was detected. Next, genes controlling immune response were upregulated. In particular, the gene encoding the IL-17 receptor was overexpressed in the RD group which is a novel finding. This should be emphasized because IL-17-related signaling can potentially be targeted, providing the rationale for future research. Establishing the molecular background associated with long-lasting and profound response to LEN may improve LEN-based chemotherapy regimens and facilitate the development of adjuvant therapies to enhance its anti-MM activity.

## Introduction

Multiple myeloma (MM) is characterized by infiltration of the bone marrow by malignant plasma cells, usually secreting a monoclonal protein, i.e., heavy or light chains of immunoglobulins which can be detected in serum and urine^1^. Nonetheless, it should be mentioned that nonsecretory MM contributes to approximately 3% of all cases^2^. Typically, MM originates from a pre-malignant state called monoclonal gammopathy of undetermined significance (MGUS). The typical sequence of events in myelomagenesis is as follows—MGUS progresses through smoldering MM to symptomatic MM requiring immediate treatment^3^. MM is the second most common hematological malignancy, accounting for approximately 1% of all cancers^4^. Therefore, it remains a significant challenge for healthcare systems and hematologists worldwide. Initially, MM was a disease with a poor prognosis and unfavorable clinical course. Nevertheless, gradual progress in basic and clinical research has led to substantial progress, and clinical outcomes tend to improve continuously over time^5^. There were several turning points in the management of MM patients. Probably, one of the most important milestones was the rediscovery of thalidomide (THAL). Later, this compound became a first-in-class immunomodulatory drug (IMiD). Based on its chemical structure, next generation molecules have been developed. Currently, there are two THAL derivatives, lenalidomide (LEN) and pomalidomide (POM), which are widely used in the treatment of newly diagnosed and relapsed MM^6^ in various treatment regimens^7,8^. LEN is probably the most widely used IMiD, and it is reasonable to assume that the vast majority of MM patients were or are exposed to this compound. However, despite very-well established efficacy, over time, patients become resistant to the drug and eventually relapse^9^. Drug-resistance is a serious challenge in modern oncology. It is estimated that as many as 90% of cancer treatment failures result from acquired resistance to chemotherapy^10^. Numerous chemotherapy resistance mechanisms common to various types of cancer have been identified. Cree and Charlton thoroughly reviewed the mechanisms by which malignant cells evade anticancer therapy and assigned them to six categories: (i) alteration of drug targets, (ii) expression of drug pumps, (iii) expression of detoxification mechanisms, (iv) reduced susceptibility to apoptosis, (v) increased ability to repair DNA damage, and (vi) altered proliferation. It should be noted that various mechanisms associated among others with tumor microenvironment and immune system also play a role^11^. Drug resistance in cancer is complex and multifaceted. Although different malignancies share the above-mentioned therapy evasion mechanisms, due to the heterogenous nature of neoplasms, there are also tumor-specific alterations mediating the development of drug resistance. Various mechanisms of resistance to LEN have been identified. The most important are CRBN downregulation or knockdown, CRBN mutations, alteration in surface antigens in MM cells, and epigenetic alterations. These and other less prominent mechanisms were thoroughly reviewed by our team elsewhere^12^. Due to its high clinical relevance, drug resistance in MM is the subject of robust and ongoing research. However, much less is known about predictors of good response to LEN-based treatment. Once established, they may be clinically relevant and beneficial in terms of patient outcomes. First, if predictors of good response to LEN were identified in patient histories and routinely performed laboratory, genetic, and cytogenetic analyses, it would be possible to stratify people with MM according to their potential response to the drug and then assign them to the optimal treatment regimen. Second, establishing the molecular background associated with long-lasting and profound drug response may further contribute to the improvement of LEN-based chemotherapy regimens and facilitate the development of adjuvant therapies that could enhance its anti-MM activity. From the standpoint of patients’ benefit such research approach is vital, as the quality of response in MM patients who received LEN is positively correlated with progression free survival (PFS) and overall survival (OS)^13^. Although there have been several reports on clinical factors predicting satisfactory outcome in MM patients receiving LEN-based treatment regimens^14–16^, there is a shortage of studies investigating the background of a good and deep response to LEN at the molecular level. There is another aspect to this approach. Many MM patients treated with LEN eventually relapse^12^. Long-term exposure to LEN induces epigenetic changes in MM cells^17^ which in turn may change the expression of various genes being potential targets for therapy. If LEN exposure were to activate alter the activity of some molecular pathways in MM cells, after relapse, the next treatment regimen could be adapted to the new genetic landscape and patients would receive more personalized treatment. Currently, it is unclear how to optimally manage LEN-resistant patients^18^. Therefore, it is critical to enhance the anti-MM properties of LEN to prolong PFS by adding potent adjuvant therapies to the LEN-containing chemotherapy regimen. Furthermore, it seems reasonable to identify potentially targetable molecular pathways to apply the best available treatment regimen after the disease progression.

In view of these premises, the aims of the study were to: (i) establish predictors of long-term and profound response to lenalidomide at the molecular level; (ii) identify alterations in gene expression and in the activity of various molecular pathways elicited by long-lasting exposure to LEN; (iii) recognize potentially targetable pathways induced by long-term exposure to LEN to select the best treatment regimen after relapse or even to provide research rationale for new treatment regimens in the future.

## Materials and methods

### Subjects and initial management

The study was conducted among patients with MM enrolled at the Department of Bone Marrow Transplantation, Pomeranian Medical University in Szczecin, Poland. The control group (Table 1) consisted of patients with newly diagnosed MM (n = 8), and the study group was treated with lenalidomide and dexamethasone (RD) (n = 8). Patients in the study group achieved at least very good partial response (VGPR). The patients were both female and male (ratio 9:7), the mean age of patients in the newly diagnosed MM group was 67 years and in the RD group 72 years, with no history of other cancers. Mean number of RD cycles in the study group was 26.63. All recruited patients in the MM control group were pre-treatment at the time of bone marrow collection. Before enrollment in the study, all patients signed informed consent in accordance with the Declaration of Helsinki (KB-006/25/23).

**Table 1:** Characteristics of the study group.

Clinical characteristics	N/mean (median); IQR
Sex F/M	4/4
Age	72.25 (73), 68.75–75.75
Hypertension NO/YES	1/7
DM NO/YES	7/1
IHD NO/YES	8/0
CKD NO/YES	7/1
Liver disease NO/YES	8/0
Respiratory disease NO/YES	8/0
Hematologic response	
CR	3
VGPR	5
Number of RD cycles	26.62 (15), 9.75–26.75
Cytogenetic risk	
Standard	8
High	0
Disease stage	
R-ISS I	6
R-ISS II	1
D-S IIA*	1

N, number; IQR, interquartile range; F, female; M, male; DM, diabetes mellitus; IHD, ischemic heart disease; CKD, chronic kidney disease; CR, complete remission; VGPR, very good partial remission; RD, lenalidomide and dexamethasone treatment regimen; R-ISS, Revised International Staging System;

*One patient was classified according to D-S (Durie-Salmon) criteria.

### Immunomagnetic separation

Bone marrow (~ 12 mL) was collected from all patients from the posterior iliac crest into tubes containing EDTA. In order to obtain a population of CD138^+^ cells from the bone marrow, an immunomagnetic isolation method was used. For this purpose, the CD138 MicroBeads kit, human (Miltenyi Biotec, Auburn, AL, USA) was used and the manufacturer’s protocol was followed.

### RNA extraction

RNA was isolated from CD138^+^ cells from each patient using the RNAqueous-Micro Kit (Thermo Fisher, Waltham, MA, USA) according to the manufacturer’s recommendations. RNA concentration and quality were measured using TapeStation 4510 (Agilent Technologies, Santa Clara, CA, USA).

### mRNA-seq

The Illumina Stranded mRNA Prep kit (Illumina, San Diego, CA, USA) and IDT for Illumina RNA UD Indexes Set A, Ligation (Illumina, San Diego, CA, USA) were used for global gene transcription analysis. 500 ng of RNA from each sample was used to prepare the library. The final loading library concentration was 1.2 pM. Sequencing parameters were in accordance with the manufacturer’s recommendations (read length: 2 × 75 bp; read type: paired end; dual index reads; the number of cycles per index read was 10). Sequencing was carried out on a NextSeq 550 instrument (Illumina, San Diego, CA, USA) using NextSeq 500/550 High Output Kit v2.5 (150 cycles) reagents.

### qRT-PCR

In order to validate the NGS method, the expression level of selected genes was measured using qRT-PCR. The genes were selected based on NGS results that appear to be important in the development of the body’s response to lenalidomide. Primers for each gene were designed using the BLAST program (sequence is shown in the Table 2). 0.1 µg of total RNA was used for reverse transcription. Reactions were performed using a First Strand cDNA Synthesis Kit (Thermo Fisher Scientific, Waltham, MA, USA). qPCR reactions were performed using the SYBR Green PCR Master Mix kit (Bio-Rad, Hercules, CA, USA). The *GAPDH* gene was used as the endogenous control gene. CFX Maestro Software (Bio-Rad, Hercules, CA, USA) was used to analyze gene expression using the comparative Ct quantification (2ΔCt) method.

**Table 2:** Primer sequence used in the qRT-PCR reaction.

Gene	Primer sequences
*FOS*	F 5′-GAGCTGCAGTGGATGGTACA -3′ R 5′-CTCCGGGCTGATCTGTTCAC-3′
*IL17RA*	F 5′-TTGCTTTGAGCACATGCACC-3′ R 5′-GAACCAGTACACCCACAGGG-3′
*IL18RA*	F 5′-ATGTGGAGCTGAACCCAAGG-3′ R 5′-GCCATGTCTGCTTTTCTCACC-3′
*FGR*	F 5′- GCAATGTTTCTGGCAGGGTG-3′ R 5′- GGGATGTGGGCAAATGAGGA-3′
*TLR4*	F 5′-GATAGCGAGCCACGCATTCA-3′ R 5′-TTAGGAACCACCTCCACGCA-3′
*FUT7*	F 5′-CACAGAGGTCAGGAAGCAGG-3′ R 5′-TTTATTGTCCACCGCCCTCC-3′
*HCK*	F 5′-GCCCAGGATGGGGTGCAT-3′ R 5′-AGGGATCTCCCAGGCATCTT-3′

### Bioinformatics analysis

Bioinformatics analysis was performed in the R environment. Genes with a fold change greater than 2 or less than − 2 were considered differentially expressed genes (DEGs) (*p* < 0.05). A detailed description of the procedures can be found in our previous article^19^.

### Statistical methods

Arithmetic means and standard deviations (SDs) were calculated using MS Excel. The analysis of statistical significance was performed using the Student’s t-test for normal distribution and the Mann–Whitney test for not normally distributed data (*p*-value < 0.05).

## Results

### Genetic landscape differs between LEN responders and newly diagnosed MM patients

**Fig. 1:**
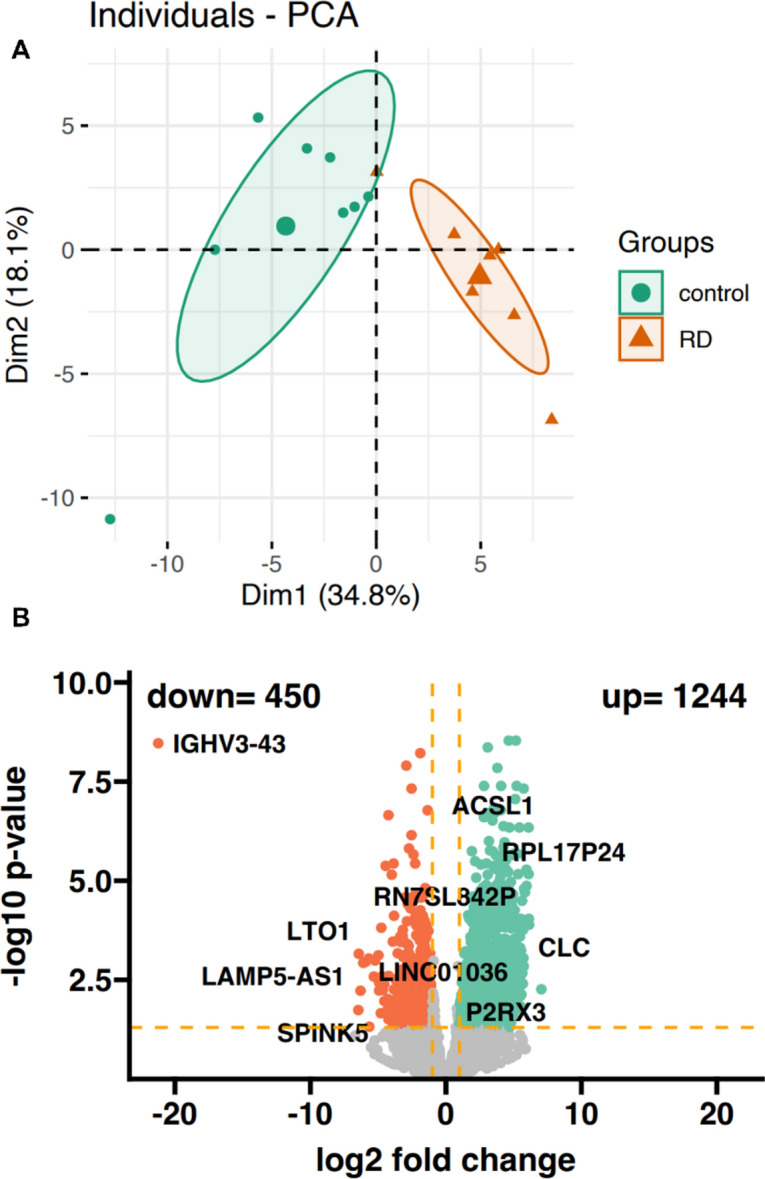
(**A**) Principal component analysis—variable graph. PCA reveals a different genetic pattern between NDMM patients and MM subjects who achieved a deep and long-lasting response to LEN. Differently expressed genes contribute to 52.9% between groups. (**B**) 1244 statistically significantly upregulated and 450 downregulated genes were detected. PCA—principal component analysis; RD—lenalidomide and dexamethasone regimen; control—NDMM; down—downregulated genes; up—upregulated genes.

Principal component analysis (PCA) revealed a different genetic pattern between control samples obtained from patients with newly diagnosed multiple myeloma (NDMM) and individuals with deep and long-lasting response to LEN. Overall, these differences accounted for 52.9% variability between the groups (Fig. 1A). This suggests that alterations in gene expression pattern are not the only key factors mediating good response to LEN. Overall, 1964 differentially expressed genes (DEGs) were detected. More precisely, 1244 upregulated and 450 downregulated genes were found in the study group compared to control samples (Fig. 1B).

### Effect of LEN exposure on the immune response and bone marrow microenvironment

**Fig. 2:**
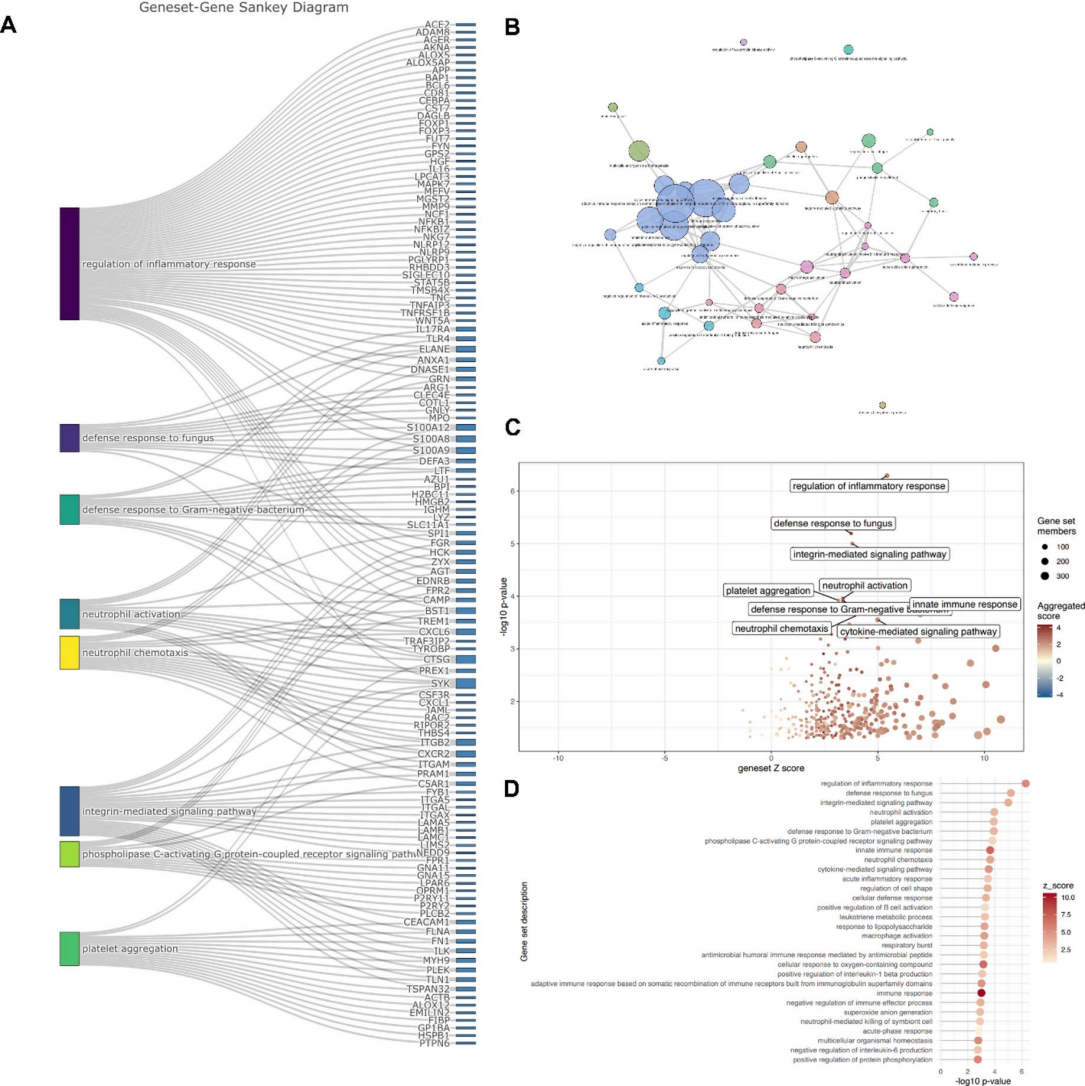
Transcriptome profiling analysis obtained by next-generation sequencing (NGS) of RNA. (**A**) Sankey diagram demonstrating functional descriptions of biological processes classified according to the Gene Ontology (GO) dataset. (**B**) Bipartite graph (Gene-Geneset panel from GeneTonic^63^ package)) assigning gene sets to their respective components. The color represents the given gene set and the size of the bubble represents the number of genes upregulated within the gene set (gene set size) according to the rule that the larger the bubble, the greater the number of genes upregulated. (**C**) Gene set volcano plot. The size of the points represents the number of genes involved, i.e., the gene set size. (**D**) Gene set description. Panels A–D were generated with Genetonic (R/Bioconductor package)^63^.

RNA sequencing revealed significant upregulation of genes controlling various processes related to both the adaptive and innate immune response (Fig. 2A–D). Gene set analysis revealed that the vast majority of upregulated genes could be assigned to the regulation of inflammatory response according to the Gene Ontology (GO) classification (Fig. 2A). Nonetheless, it should be noted that a particular gene may be involved in more than one functional annotation (Fig. 2A). Moreover, analysis of the bipartite graph (Fig. 2B) and gene set description (Fig. 2D) revealed that IL-1 is positively regulated, whereas IL-6 negatively regulated in the study group. Other findings include the involvement of processes classified as “integrin-mediated signaling pathway” and “regulation of cell shape”. These findings suggest that LEN treatment induces changes in multiple immune processes and also alters interactions between MM cells and the surrounding stroma, i.e., the MM niche and the bone marrow microenvironment.

To shed more light on the exact role of the mentioned changes, gene expression analysis was performed.

### Gene expression pattern associated with good response to lenalidomide

Globally, 1244 upregulated and 450 downregulated genes were detected in the study group compared to control samples (Fig. 1B). Most of them could be assigned to functional annotations implying the involvement of the bone marrow microenvironment as well as immune responses. Therefore, alterations in these processes and changes in expression of the respective genes can be elicited by therapy. Thus, heatmaps were created to precisely identify gene expression patterns and molecular pathways that could serve either as predictors of satisfactory response to LEN or as potential therapeutic targets for agents that could thereby enhance LEN activity against MM.

First of all, it should be noted that green is the dominant color in all the figures presented in the studied group. Therefore, it can be hypothesized that LEN causes a global increase in gene expression in people responding to therapy. Although the analysis presented does not provide definitive answers about the underlying mechanisms, there are some putative explanations that will be discussed later. It is important to note that we analyzed gene expression patterns in plasma cells, which, although malignant, are highly specialized. Hence, genes that may be considered relevant for further analysis and discussion are supposed to be expressed in plasma cells or malignant cells in general. Genes that have been shown to be associated with good prognosis or to be essential for tumor progression but were nevertheless shown to be expressed in surrounding cells (tumor niche) rather than in the tumor cells themselves were excluded from the analysis.

#### Integrin-mediated signaling pathway and regulation of cell shape—interactions within MM niche

**Fig. 3:**
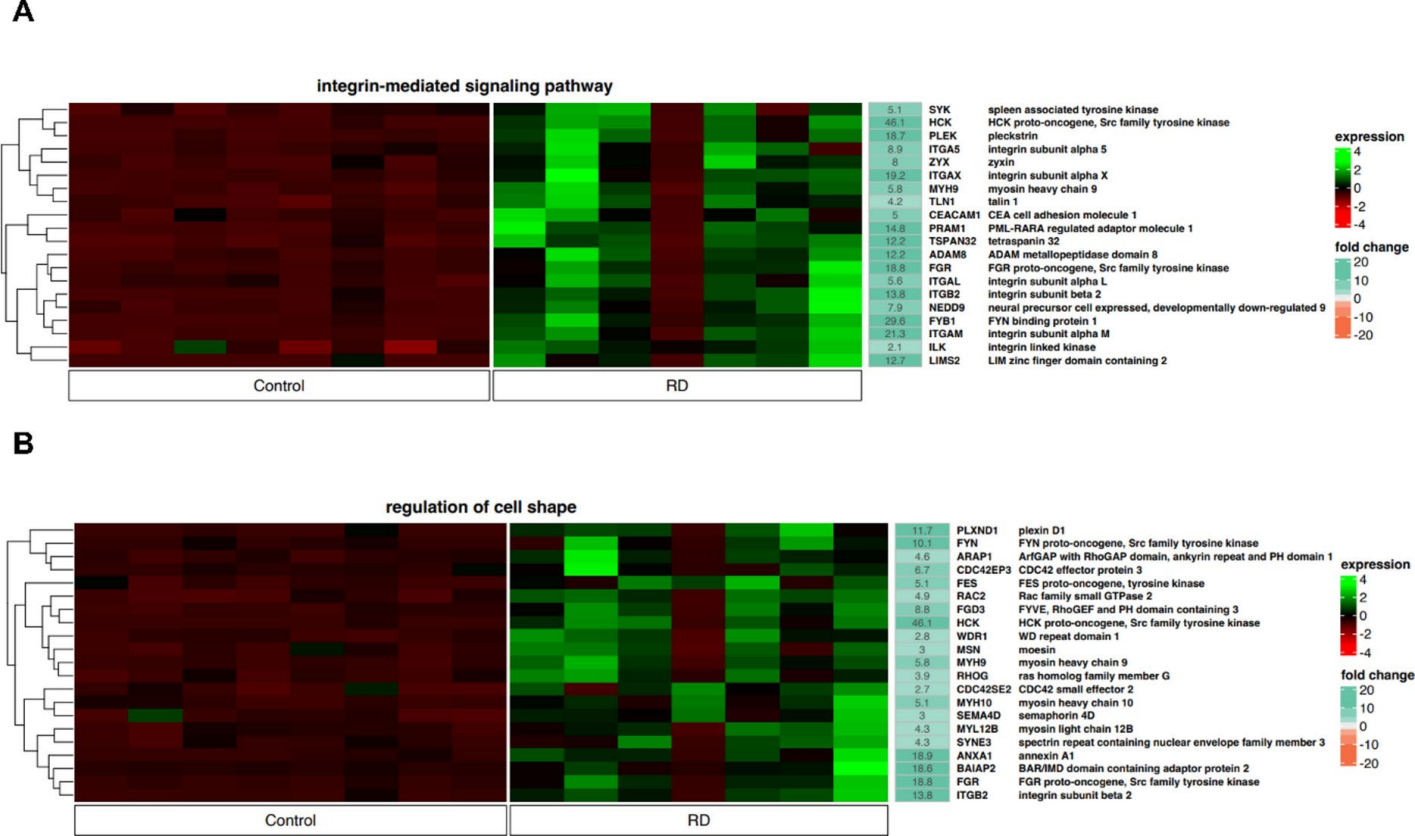
Heatmaps demonstrating the expression of genes controlling processes involved in interactions within the MM niche and the bone marrow microenvironment. (**A**) Integrin-mediated signaling pathway (20 genes) and (**B**) regulation of cell shape (21 genes). Genes were assigned to particular functional annotations according to the GO classification. The color scale depicts the gene expression level from low to high (dark red to green, respectively) and the fold change from low to high (orange to sea green). Control—newly diagnosed multiple myeloma; RD—multiple myeloma patients treated with lenalidomide and dexamethasone.

Our analysis revealed genes involved in regulation of interactions between cells and the cellular microenvironment, including interactions within bone the marrow microenvironment^20^ and tumor or, in this particular case, MM niche^21,22^. Identified genes are assigned of “integrin-mediated signaling pathway” and “regulation of cell shape” according to the GO classification of functional annotations (Fig. 3A and B). Globally, upregulation of virtually all genes controlling the above-mentioned GO functional annotations is observed in patients who have achieved a good response to LEN. Surprisingly, it should be emphasized that most of the genes with increased expression are involved in the growth and progression of cancer, including MM. As example could be presented genes from the “integrin-mediated signaling pathway”: *HCK*^23,24^*, FYB1*^25^, *PLEK*^26^, *ITGAM*^27^ and *FGR*^24^ (Fig. 3A). “Regulation of cell shape” (Fig. 3B) could be exemplified by *HCK*^23,24^, *ANXA1*^28^, *FGR*^24^, *ITGB2*^29^. These genes were selected due to their high, over 15-fold change. Their presumed role will be discussed below. On the contrary, in NDMM the global downregulation of identified genes is by all means apparent.

#### Modulation of immune system reflects the response to Rd treatment regimen

**Fig. 4:**
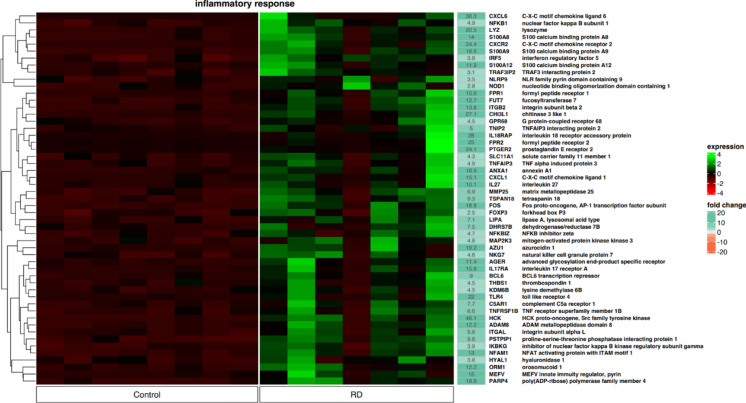
Heatmap showing the expression of genes controlling processes involved in inflammatory response according to the GO classification (53 genes). The color scale depicts the gene expression level from low to high (dark red to green, respectively) and the fold change from low to high (orange to sea green). Control—newly diagnosed multiple myeloma; RD—multiple myeloma patients treated with lenalidomide and dexamethasone.

Furthermore, the analysis identified genes involved in the regulation of the “inflammatory response” according to the GO classification (Fig. 4). In the control group, the identified genes were significantly downregulated, while in the LEN responder group, global upregulation is clearly visible. 21 genes with fold change greater than 10 were identified. Among them, there were 15 genes with a fold change greater than 15. Although no definite clusters were identified, in MM individuals receiving RD regimen global gene upregulation was detected.

We found that some of the upregulated genes assigned to the “inflammatory response” are associated with good clinical outcomes in cancer patients. However, it should be noted that there are several genes that, according to various studies, are bound to cancer progression. Overall, the genetic landscape is heterogenous. More specifically, it is a combination of genes that have been shown to be associated with good prognosis and response to treatment, as well as with cancer progression and poor survival.

**Fig. 5:**
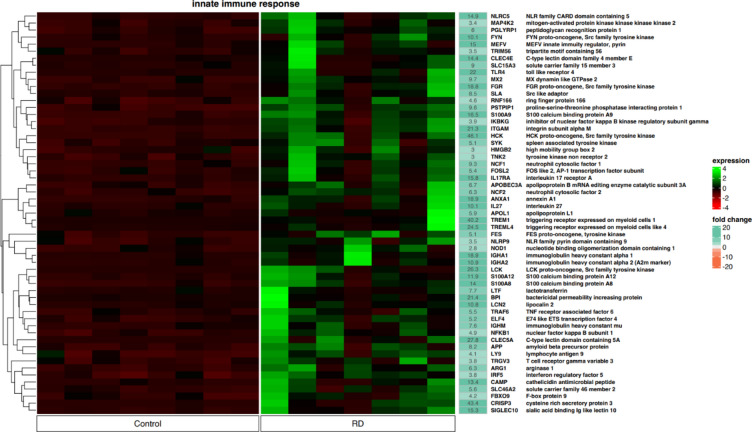
Heatmap showing the expression of genes controlling processes involved in the innate immune response according to the GO classification (57 genes). The color scale depicts the gene expression level from low to high (dark red to green, respectively) and the fold change from low to high (orange to sea green). Control—newly diagnosed multiple myeloma; RD—multiple myeloma patients treated with lenalidomide and dexamethasone.

In addition, significant changes in the expression of genes involved in the regulation of the “innate immune response” according to the GO classification were revealed be the analysis (Fig. 5). In the control group, the identified genes were significantly downregulated, while in the LEN responder group, global upregulation is clearly visible. More specifically, 25 genes were identified with a fold change greater than 10. Among them, there were 16 genes with a fold change greater than 15. Similar to the “inflammatory response,” no specific clusters were identified, but global gene upregulation was detected in MM subjects receiving the RD regimen. It should be noted that some genes overlapped and were detected in both functional annotations.

**Fig. 6:**
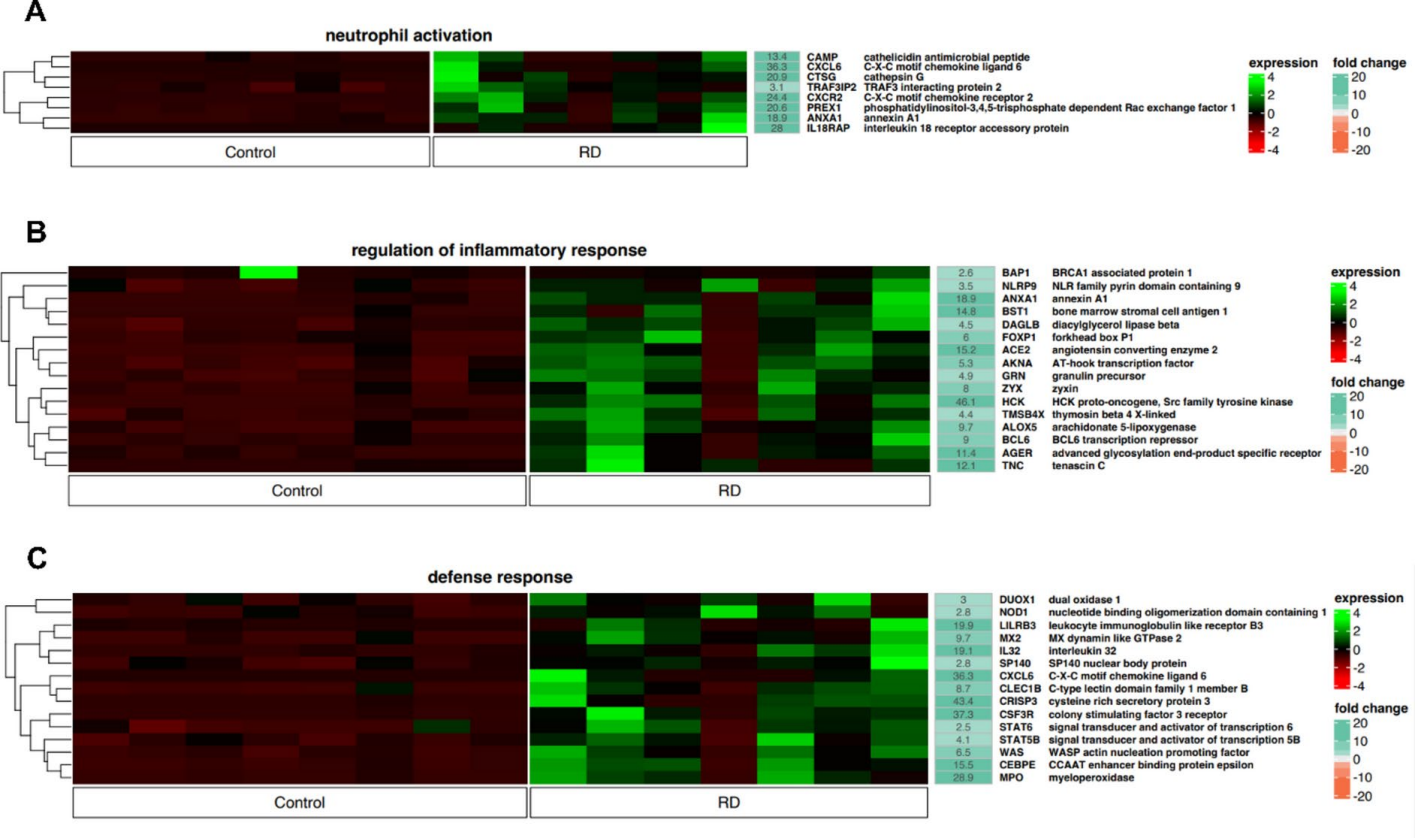
Heatmap showing the expression of genes controlling processes involved in (**A**) neutrophil activation (8 genes) (**B**) regulation of inflammatory response (16 genes) (**C**) defense response (15 genes). Genes were assigned to individual functional annotations according to the GO classification. The color scale depicts the gene expression level from low to high (dark red to green, respectively) and the fold change from low to high (orange to sea green). Control—newly diagnosed multiple myeloma; RD—multiple myeloma patients treated with lenalidomide and dexamethasone.

Altered expression of genes involved in the regulation of “neutrophil activation”, “regulation of inflammatory response”, and “defense response” according to the GO classification distinguishes LEN responders from NDMM (Fig. 6A–C). In the control group (NDMM), the identified DEGs were significantly downregulated, while in the LEN responder group, global upregulation was clearly visible. More precisely, 20 genes were identified with fold change greater than 10. Among them, there were 16 genes with a fold change greater than 15. As in previous functional annotations, no definite clusters were identified but in MM individuals receiving RD regimen global gene upregulation was detected. It should be noted that some genes overlapped with each other and were detected in other previously analyzed functional annotations. The identified genes included DEGs that are presumed to influence good response to therapy or are associated with poor prognosis and disease progression. Interestingly, some DEGs or their downstream pathways can potentially be targeted, making them potentially clinically relevant.

### Upregulation of IL-17RA—a potential therapeutic target

**Fig. 7:**
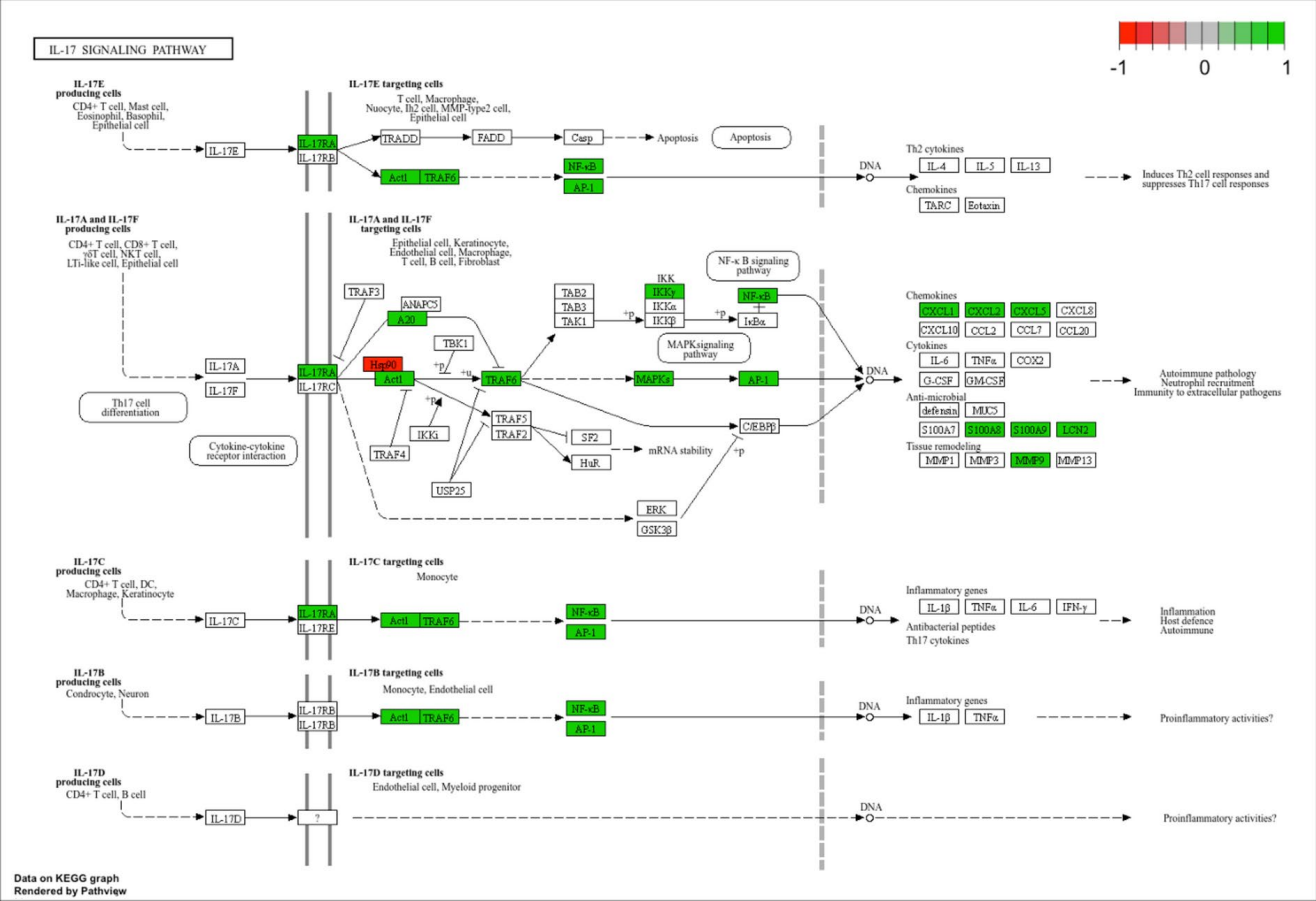
IL-17 signaling pathway in CD 138 + MM cells. Green—statistically significant increase in expression, red—statistically significant decrease in expression. IL-17 signaling pathway was obtained from The KEGG pathway database^64–66^.

Overall, the analysis revealed 1964 differentially expressed genes (DEGs). Among them, 1,244 genes were upregulated and 450 were downregulated. The genes were analyzed for possible clinical significance and putative therapeutic intervention. All genes with a fold change in expression greater than 10 were analyzed in detail. We found that the gene encoding the IL-17 receptor (IL-17RA) was significantly upregulated (Figs. 4 and 5) with a 15.8-fold change in expression. IL-17RA elicits molecular actions of IL-17 within MM cells. In addition to the upregulation of IL-17RA, the bioinformatical analysis (Fig. 7) revealed increased expression of NF-κB and MAPK, which are key molecules in IL-17 signaling pathway^30,31^. From a clinical standpoint, IL-17 is a growth factor for plasma cells, including malignant MM cells^32,33^. Furthermore, IL-17 mediates SMM-to-MM transition, MM progression^33^ and extramedullary disease^34^. In addition, IL-17 is associated with bone disease in MM^35^ and may be considered a negative predictor of MM prognosis^36^. Since monoclonal antibodies directed against IL-17 or IL-17R are commercially available, this finding has potential clinical implications.

### qRT-PCR validation of selected genes

Following good laboratory practice we validated our results with qRT-PCR. Validation confirms data obtained from RNA sequencing (Table 3). The above genes were selected as they play a role in cancer biology or are potentially targetable. Their potential relevance is discussed below.

**Table 3:** Validation of selected genes with qRT-PCR.

Gene	NDMM [mean; ± SD]	RD [mean; ± SD]	Fold change in expression	*p*
*FOS*	99.65 ± 85.5	**3673.27 ± 2403.3**	16.8	0.00214
*IL17RA*	102.26 ± 98.5	**364.58 ± 127.4**	15.8	0.00496
*IL18RAP*	0.44 ± 0.42	**6.77 ± 6.4**	28	0.00328
*FGR*	1.20 ± 1.4	**14.90 ± 14.3**	18.8	0.01046
*HCK*	16.12 ± 22.0	**40.46 ± 21.0**	46.1	0.03846
*TLR4*	25.26 ± 23.1	**384.86 ± 250.8**	22	0.00214
*FUT7*	9.73 ± 13.8	**131.72 ± 98.7**	12.7	0.00214

NDMM, newly diagnosed multiple myeloma (control group); RD, multiple myeloma patients treated with lenalidomide and dexamethasone (study group).

Bolded results showed statistical significance (*p* < 0.05).

## Discussion

LEN is a potent anti-MM agent that is increasingly used worldwide^18^. It can be hypothesized that virtually all MM patients were or are exposed to the drug. Therefore, a deeper understanding of the mechanisms of LEN resistance, predictors of deep and long-lasting response, and alterations in gene expression and activity of various molecular pathways elicited by LEN is of paramount importance. Drug resistance mechanisms are currently the subject of ongoing research. However, clinical and genetic factors predicting good drug response as well as changes in various molecular pathways induced by drug exposure are not well explored. Currently, it is unclear how to manage LEN-resistant patients^18^. Therefore, it was important to conduct this study and shed more light on LEN-induced changes in MM cells. Identification of these pathways may provide rationale for adjuvant therapies that enhance the anti-MM properties of LEN and prolong PFS. Moreover, the recognition of potentially targetable molecular pathways may help in administering optimal treatment regimen after the disease progression and may provide a rationale for further studies investigating novel treatment regimen for individuals exposed to LEN.

The aim of this preliminary study was to establish the predictors of long-term and profound response to LEN at the molecular level and search for potential clinical implications.

First, we identified upregulation of genes involved in interactions with the bone marrow microenvironment and, especially, with MM niche. The role of the bone marrow niche in MM is very well established and does not require much explanation. Briefly, it creates a favorable environment and promotes disease progression^37^. Furthermore, upregulated genes are associated with cancer progression, including MM. For instance, we can list the following genes: *HCK*^23,24^*, FYB1*^25^, *PLEK*^26^, *ITGAM*^27^, *FGR*^24^, *ANXA1*^28^, *ITGB2*^29^ (Fig. 3).

However, we analyzed samples obtained from patients with a long-lasting and deep response to LEN, with no signs of a relapse. To the best of our knowledge, this is the first observation and probably a novel finding reporting upregulation of genes and processes associated with interaction with the tumor niche during LEN treatment. LEN not only interacts with MM cells, but also affects various cells in the bone marrow, affecting MM niche^38,39^. We demonstrated that despite deep and long-lasting response, genetic profile suggests that tumor niche remains, at least partially, permissive to MM. In this case, we came up with two potential explanations. Firstly, the upregulation of these genes may be exemplification of the effort by which the remaining MM cells attempt to evade the anti-MM properties of LEN associated with their interaction with the MM niche and could be considered a type of negative feedback. Secondly, patients were on RD regimen. Perhaps the combination of LEN and dexamethasone alone is not strong enough to change the bone marrow microenvironment to be less favorable to the disease. Nevertheless, it can be hypothesized that the MM niche and the internal interactions are the cause of relapse and progression. This further strengthens existing evidence that the bone marrow microenvironment should be the primary target of anti-MM therapy. In fact, the most important anti-MM agents, whether anti-CD38-based therapies, iMiDs, CAR T-cell therapies, bispecific antibodies and antibody–drug conjugates, preferentially target the bone marrow niche and constitute the current therapeutic standard^39^.

Subsequently, we identified the upregulation of genes involved in immune system modulation, which were assigned to “inflammatory response”, “innate immune response”, “neutrophil activation”, “regulation of inflammatory response”, and “defense response” according to GO functional annotations. Global results showed an increase in gene expression compared to control samples from NDMM patients. This time the genetic landscape is more heterogenous, and according to the literature, upregulated genes are associated with both cancer progression and poor prognosis, as well as chemosensitivity and favorable prognosis. For instance, the *LYZ* gene has been shown to be significantly downregulated in MM cells compared to normal plasma cells^40^. In our study, we detected a 20.5-fold change in expression, suggesting that long-lasting exposure to LEN restores the expression of this gene and makes these cells more like their non-malignant counterparts. In addition, we demonstrated an increase in the expression of *FPR1/2* genes, which have been shown to be essential for chemotherapy-induced anticancer immune responses^41^. We also demonstrated an increase in the expression of various genes, such as *AZU1,* which is considered as tumor suppressors^42^. *NLRC5* overexpression, which was detected in LEN responders, may be a hallmark of chemosensitivity in MM. According to the study by Yoshihama et al. in most tumors, NLRC5 levels were decreased in malignant cells compared to normal tissue. It should be also emphasized that lower *NLRC5* expression was associated with reduced 5-year survival in melanoma, rectal, bladder, uterine, cervical and head/neck cancer, suggesting a prognostic value of *NLRC5* expression levels^43^. On the other hand, we detected upregulation of genes whose role in malignancy is either ambiguous or strongly negative and associated with unfavorable clinical outcome. In the first place, our results revealed the upregulation of other genes that are associated with tumor growth and progression in various cancers. For instance, CHI3L1 has been shown to be involved in enhancing the metastatic potential of gastric cancer^44^, FUT7 promotes epithelial-to-mesenchymal transition in bladder urothelial carcinoma^45^ and is associated with drug resistance and inferior PFS in MM^46^. In addition, FOS mediates LEN resistance in multiple myeloma^47^ and its upregulation may be another attempt to evade its anti-MM activity. Another upregulated gene was *HCK*, a member of the SRC family. This gene has been proved to mediate tumor progression in colorectal cancer^24^. It is overexpressed in MM^48^, and its therapeutic inhibition represents a promising direction of research in cancer treatment^49^. In addition, we demonstrated overexpression of *PARP.* Although the exact role of PARP in MM is unknown, it is important to mention that PARP inhibitors are currently being investigated as potential anti-cancer agents^50^.

Moreover, we demonstrated an increase in the expression of genes encoding receptors for IL-18 and IL-17. This finding should be discussed in detail due to its high novelty and potential clinical implications. Moreover, it provides the rationale for further preclinical studies and even future clinical trials. High expression of *IL18RAP*, a gene encoding receptor for IL-18 has been shown to positively affect OS in HCC (hepatocellular carcinoma) patients^51^. Moreover, it exhibits antitumor effects in colorectal cancer^52^. On the other hand, it has been shown that IL18 promotes the growth and progression of MM, and high levels of this cytokine in the bone marrow are associated with a poor prognosis in MM patients^52^. Although IL-18 appears to have beneficial effects in solid tumors, in MM rather is a hallmark of bad clinical outcome. Overexpression of *IL18RAP* may be an attempt to increase IL-18-related signaling and thus avoid anti-MM effects in LEN. Therefore, the role of IL18RAP requires greater attention and further research as it may become a potential target for new classes of anti-MM agents.

Another significantly upregulated gene in the study group was *IL17RA,* which encodes the receptor for IL-17. IL-17 receptors (IL17R) have been shown to be expressed on plasma cells^32^. Furthermore, IL-17 promotes the growth of MM cells both in vitro and in vivo via IL-17R and hinders immune response in this condition^53^. Several other studies support the role of IL-17 in the growth and progression of MM^54^. Moreover, IL-17-related signaling has been shown to be involved in bone disease in MM^35^. Once IL-17 binds to its receptor, its molecular actions begin via the activation of downstream mechanisms. Briefly, the most important signaling pathways include the activation of the canonical NF-κB and mitogen-activated protein kinase (MAPK) pathways^30,31^. Our results confirmed that both NF-κB and MAPK are upregulated in the study group (Fig. 7) implying that interference with IL-17 axis my enhance the anti-MM activity of LEN. Indeed, according to existing studies, interfering with IL-17 signaling in MM could be considered as a potential therapeutic approach. For instance, Prabhala et al. showed that a monoclonal antibody against IL-17A reduced tumor growth and reduced bone damage in a mouse model of MM^55^. In light of these premises, it seems reasonable to consider IL-17-related signaling as a new potential therapeutic target in MM. Our results demonstrated upregulation of gene encoding IL17R in MM cells exposed to LEN and this is probably the first report revealing the upregulation of this gene induced by long-lasting exposure to LEN. The exact meaning of *IL17RA* upregulation remains to be elucidated. Nevertheless, it can be hypothesized that this may be an attempt to increase IL17-related signaling in MM cells and overcome the anti-MM effects of LEN. Whatever the reason, this discovery opens up new options and directions for research. To begin with, it should be mentioned that there are monoclonal antibodies against IL-17, namely Ixekizumab, Secukinumab or its receptor, Brodalumab, which have been successfully implemented in the treatment of various dermatological and rheumatoid diseases^56^. Although IL-17 inhibitors are relatively safe, they increase the risk of non-severe infections^57^. It should be mentioned that they do not have the proclivity to induce cytopenia^58^, which is a very common adverse reaction of anti-MM agents^59,60^, often resulting in dose modification or even discontinuation of treatment^60^. Therefore, the combination of LEN and IL-17 or IL-17R inhibitors seems to be a worthy research direction to explore, at least in the following scenarios. First, they could be added to LEN-based treatment regimens as an adjuvant therapy because LEN exposure appears to increase IL-17R expression. Second, given that LEN induces IL-17R expression in MM cells, IL-17 inhibitors, especially Brodalumab, could be tested sequentially after disease relapse when the patient becomes refractory to LEN as part of a subsequent treatment regimen. To the best of our knowledge, this is a novel finding and is the first study reporting increased expression of the gene encoding IL17R induced by LEN.

## Conclusions

Long-term exposure to LEN changes the genetic footprint in MM cells. We detected overexpression of genes associated with both good response to treatment and tumor growth and progression in MM cells isolated from bone marrow samples obtained from patients with at least VGPR. Upregulation of genes that have been shown to mediate drug resistance and disease progression may be an attempt by residual MM cells to evade cytotoxic effects of LEN. Some of the upregulated genes and their associated molecular pathways, with particular emphasis on IL17RA, can potentially be targeted, opening new research opportunities.

### Study limitations

Our study yielded interesting and novel results. Nonetheless, it has some drawbacks. First, due to the high costs of NGS and available kits we included representative samples obtained from only 8 Rd and 8 NDMM patients. Subsequently, RD patients achieved at least VGPR, so the number of MM cells in the bone marrow was very low. We managed to isolate RNA using highly sensitive kits, but we were unable to isolate DNA and conduct methylation analysis, although the gene expression profile implies involvement of epigenetic mechanisms. Moreover, patients in the study group received LEN and dexamethasone, therefore it might be difficult to distinguish which compound is responsible for upregulation of gene encoding IL-17RA. Nonetheless, having considered that steroids, including dexamethasone, inhibit IL-17 related signaling^61,62^ it might be concluded that LEN accounts for majority of this effect.

## Data Availability

Data are available upon request form the correspondence authors.
